# A Present Menace to Education1Opening address of the Medical Department of the University of Buffalo at the beginning of the sixty-fourth annual session, September 27, 1909.

**Published:** 1910-06

**Authors:** P. W. Van Peyma

**Affiliations:** Buffalo, N. Y.; Associate Professor of Obstetrics University of Buffalo


### BUFFALO MEDICAL JOURNAL.

VOL. LXV. JUNE, 1910. No. 11

### ORIGINAL COMMUNICATIONS.

### A Present Menace to Education.[1]

BY P. W. VAN PEYMA, M.D., Buffalo, N. Y.
Associate Professor of Obstetrics University of Buffalo

THE subject, to which your attention will be briefly called, might be entitled "A Present Menace to Education." That we are living in an age of most rapid evolution—at times appearing even revolution—is recognised by all. Discoveries follow discoveries, development follows development with a rapidity of succession that is startling; satisfying even the most devoted lover of sensation. This has continued until the very atmosphere is charged with it, and men and women breathe the exhilarating, stimulating air. We are intoxicated with the rapid succession of events, we are feverish in expectation of new ones. So many happenings are crowded into the briefest space, that time appears to have become more and more precious. We feel that we must hurry; hurry to keep up with events. We must keep informed; we must know what is happening,—what is next to happen.

During the last fifty years more has come to pass than has ordinarily occurred in ten times that period, and the rate is constantly increasing. To parallel this in our organisms, to keep up with this headlong race, our breathings should be, at least, one hundred and fifty, our heart beats several hundred a minute, we should never think to exceed in sleep, one hour in the twenty-four. But the healthy human organism refuses to follow this breakneck pace. The capacity of the human mind continues about that of the times of Plato and Euclid. Yet science continues to expand and to differentiate. New subjects of study and interest are continually being brought forth. Each has its claims to importance and recognition, and all are so correlated that none can be thoroughly studied singly.

The curricula of institutions of learning are crowded, and teachers are at a loss how to handle this ever-increasing fund of knowledge. Methods and systems of teaching have been devised

1. Opening address of the Medical Department of the University of Buffalo at the beginning of the sixty-fourth annual session, September 27, 1909.

to more expeditiously dispose of, and, if possible, to assimilate this accumulation of data. But, at last, even the most ardent and sanguine believers in the advantages and possibilities of improved methods begin to recognise that the undertaking is beyond them, at least within the time prescribed. The results, as seen in imperfect education and in impaired health, have been only too apparent.

It is quite true that certain surface indications may appear to prove the contrary. We have a public school system that, in many respects, is excellent. We have obligatory attendance, with truant officers to enforce attendance. A recent law provides for a complete record of all children of school age. We require entrance examination for admission to high schools and colleges, general and special. Regents' points are required, and the number has been increased from time to time. Many technical colleges are even demanding certain degrees as prerequisites to admission. In medical colleges the course has been lengthened, so that where formerly two years sufficed, now four or five years are required. But, on looking below the surface, the real conditions appear markedly less favorable. The public school course which formerly extended over ten years, has been shortened to nine, and is now practically only eight, since the ninth year is devoted to high school subjects. The recent resignation of a teacher in an adjoining city because she did not believe in the wisdom of teaching Latin in the eighth grade, seems to indicate that even the eighth grade is being invaded by high school subjects, thus still further curtailing the grammar school curriculum. And not alone has the course been shortened to practically four-fifths of its former time, but the number of subjects has been materially added to. Music, physical culture, manual training, sewing, domestic science, physiology, nature study and civics are some of the subjects which in recent years have been added to the course of study. The result is a crowding and a hurrying, where memorising is forced to take the place of careful study, reflection and mental assimilation.

For the average child the present crowded grammar school and high school curriculum means either overwork and impaired health, or what is much more common, a superficial acquaintance with, a mere smattering of a rather large number of subjects, with a thorough knowledge and understanding of none. This condition begins in the grammar school, and continues through the high school.

As an indication of how little regents' points evidence real knowledge, I may say that I have known of many children that graduated from our grammar schools, where even the present condensed and crowded curriculum had been further abbreviated by the skipping of grades. In some instances three and even four years' work was covered, or rather, slid over, in one year. It happens not infrequently that children are graduated from the grammar, high and training schools or from the grammar and normal schools at the early age of eighteen years, and at this early age, immature in every sense, become teachers.

In the high school one can often see young people taking four or five subjects at one time, with music perhaps as an additional study at home. The essential difficulty of the several subjects may be judged from the following list taken from the high school prospectus: physics, geology, psychology, botany, zoology, algebra, geometry, trignometry, astronomy, German, French, Spanish and Latin. Many of these subjects are supposed to be covered in one term.

The bearing which all this has upon the teaching in this our own institution is evident, since, while my personal knowledge of conditions is local, there is no reason for believing that conditions here do not compare well with the average. It is true, as already remarked, that in medical colleges the course of study has been noticeably lengthened, instead of shortened. But this advantage can hardly offset the want of more deliberate and thorough preliminary mental training and preparation. The want of this is not infrequently observed in more or less lack of logical reasoning power, in want of suspension of judgment pending thorough consideration; in failure to recognise the depth and the scope of a proposition.

The higher institutions of learning not alone suffer from the want of the proper mental training and equipment of their freshmen, but in not a few instances, they continue this hot house process of what might perhaps be termed "intensive" teaching. Besides it is quite the fashion nowadays to emphasize the social athletic and general cultural advantages of a college education, rather than the more homely and solid mental acquirements. The result in these cases is sometimes not particularly valuable, and it is undoubtedly true, as a well known educator, himself a college graduate, recently remarked, that those persons rate a college training highest who know least about it.

It is an old saying that a little knowledge is a dangerous thing. The superficiality of the present day, in its ignorance of what constitutes real knowledge, tends to conceit and to foolish and arrogant assumption.

Having no appreciation of the vast scope and the profound depth of the science of today, the average man, and especially he who prides himself on his socalled education, presumes to pass immediate and positive judgment on all matters of inquiry. In science, politics, finance, theology, there is nothing upon which he is not ready to give his views, and that with an assurance and a positiveness that is astonishing.

With all our vaunted diffusion of knowledge and education, there has probably never been a time when there existed so many "isms," fads and crazes; each with its multitude of followers and defenders. Perhaps there has never been a time when there was so little suspension of judgment, so much headlong jumping at conclusions. And it should be remembered, that, especially in a country living under a democratic form of government, where the masses are at the helm, is this condition one fraught with peril. Never was there a more fruitful field for demagogy, in all the activities of life. Scarcely is one of these mushroom movements on the wane, than several arise to take its place—and all this in the name of progress and reform. Conservatism is regarded as a cardinal sin, and pity and compassion are its lightest penalties.

It is, of course, not maintained that these are universal facts, showing no exceptions. But it is maintained that they represent the general tendency. If it be admitted that this is the general tendency, and these the results, the question naturally arises, along what lines may a better tendency and a better condition be brought about. Something can be said in favor both of a wide general knowledge, necessarily of limited depth; and, on the other hand, of a profounder knowledge of a much more limited scope. It is clearly impossible for even the greatest intellect to cover the whole field. Of Francis Bacon it was said, that he knew all knowledge, but, however, that may have been, it will certainly never occur, either now or in the future, that one mind will grasp all acquired knowledge. It is also clear, especially to medical men, that education requires time, and that haste means waste and failure.

In view of these facts, it is suggested that a sensible, valuable compromise would be found in a fairly general review, of a scope not too large; with the addition of a comparatively thorough study of at least one subject, of a scientific nature, and one involving a certain amount of correct reasoning. This latter study would teach thoroughness, and would naturally result in a recognition of how much there is to this particular science, and, by inference, how much there must be to the other sciences. As further results we would expect to see intellectual modesty, scientific curiosity, and an active interest in further study. If parallel with this there were also thorough training of the mind in logical thinking such a person would start out from his school days with a recognition of his short comings, and with a desire and a trained aptitude for further study, and thus give assurance of continued development throughout life. This would be true education, the education that makes good physicians, good citizens, good men and women.

A letter written several years ago, at a time when the writer was a member of the Board of School Examiners to one also interested in the public schools, calls attention to many points which are still matters of public concern.

It has seemed to me that the evident policy to cover a certain amount of ground within a definite period often works harm, and results not infrequently in a feeling of being hurried. In very many instances there seems to be altogether too little time for thorough digestion and assimilation.

The skipping of grades has become very common having recently reached its climax in a certain school where children passed in one year through three and four grades. I believe that it rarely happens that a pupil cannot remain in a grade with profit. Even though he may have mastered, fairly well, the subject matter, a competent teacher can present it in so many different lights that the subject will come to be much better comprehended. As a result of this hurry we see very many graduates from the training school who are still well in their teens. This is unfortunate and the consequence is that many of our teachers show both superficiality of knowledge and general immaturity. I am thoroughly convinced that examinations receive entirely too much attention. In the eighth and ninth grades especially, a feverish anxiety develops, with consequent cramming, and physical and mental impairment." As the time for examinations approaches teachers may be seen drilling the pupils on the questions of previous regents examination." If the regents' examinations are to be continued, it is certain that they should not receive the controlling attention which has been given them in the past. It seems clear also that undue stress is laid upon uniformity and fixity of method, leaving too little leeway for individuality of teacher and pupil.

To my mind the principle object of education is to develop the power to think logically and to reason correctly. It is this faculty which later must be brought to bear upon the problems of life and which will determine, more than any other intellectual factor, the question of future success or failure. Imbued with this conviction, I have felt that the schools are too intent upon getting certain technical results, being comparatively indifferent as to thorough comprehension of principles; and passing from one step to another, without determining that the former step is thoroughly understood. A well trained mind, when occasion requires, will rapidly acquire and master technical facts, but an untrained mind, though saturated with facts, remains always an automaton.

The development of civic virtues is another subject which receives scant attention. The study is almost entirely limited to a discussion of terms of office, manner of election, routine duties, and the like. I have read with pleasure the counsel to teachers not to "be satisfied with merely giving information, but to try to inculcate a high standard of public duty, the obligation of patriotism and civic virtue, a sense of the danger to the country arising from official corruption." That they are to show the need of placing public interests above private gain, and to insist on the principle that every office exists solely for the benefit of the people, and never for an individual or a party." I should like to see this injunction printed, hung in every class room and made the subject of regular and earnest discussion.

I am inclined to believe that the multiplicity of subjects, and this especially in the High Schools, contributes to shallowness of comprehension. Until recently physics was covered in one term, and the same is still true of geology, psychology, botany, zoology and astronomy. It would seem much better to drop several of these subjects from the curriculum than to continue to skim over them as hitherto. Something can of course be said in favor both of a broad, general knowledge, on the one hand, and of a profound knowledge of much less scope on the other. The rapid advances of science will undoubtedly necessitate an occasional modification and readjustment of curriculum. It has seemed to me, based on my own experience, that a sensible compromise might be made by insisting that at least one subject,—of a scientific nature,—shall be studied during a long period. In this way, the student will be brought to discover the relation which this subject bears to many others,—will come to recognise the correlation of all knowledge. He will also learn how much this single study involves, and, by inference, how much is comprised in the other studies, but superficially dwelt upon. He will learn to recognise the vast extent of knowledge, and the narrow scope and the superficiality of his own acquirements. This will teach him modesty, and the necessity for thoughtfulness, and carefulness of judgment. Citizens thus educated would be less impulsive, less rash, less likely to subserve the schemes of demagogues.

I shall not go into a detailed statement of the observations upon which I base my conclusions. Still it may be worth while to mention a few of the more general. It may be recalled that I noted the teaching of fractions throughout the fifth grade. Since then, I have extended the inquiry into the sixth and higher grades. In a few instances, also, I have questioned high school pupils. The results have been disappointing. I have found only about eight fifth grade rooms where the pupils were able to do the example $6 \div 2/3 =$ ?; and at the same time explain the problem, and show that they understood the principle. The result in the sixth grade was no better, and even in the higher grades it was unsatisfactory. In many instances the pupils had been taught to perform the examples by inverting the divisor and multiplying, but had not been made to understand why this gives the answer. As a result, many simply multiplied without in verting, getting the same answer for division as for multiplication. This shows that memory has been made to take the place of understanding. As Milne, in his preface to the Standard Arithmetic, says, a student who has been made to understand every step "will never forget a process or a rule, because he can devise the process and frame the rule at will."

Another fact frequently observed, is that children memorize and recite definitions without understanding the definition, or the meaning of the words employed. This has been especially noticeable in geography and grammar. For example, they will say that a preposition is a word which shows relation, without having the slightest idea as to the meaning of the expression.

In many instances where I have questioned the class as to the purpose of all the study of antecedents, clauses, phrases, subject, predicate, and the like, the replies have shown that the pupils lack a definite or clear conception of the bearing and value of all this work. This, of course, shows a failure to provide an incentive, based on apperception and interest. I think that many teachers might with profit read and study the quotation from John Stuart Mill, which heads the preface of Maxwell's Grammar, and in which he points out the relation existing between logical thought, correct expression, and grammar. "Consider for a moment what grammar is. It is the most elementary part of logic. It is the beginning of the analysis of the thinking process. The principles and rules of grammar are the means by which the forms of language are made to correspond with the universal forms of thought. The distinctions between the various parts of speech, between the cases of nouns, the moods and tenses of verbs, the functions of participles, *are distinctions in thought not merely in words.* Single nouns and verbs express objects and events, many of which can be recognised by the *senses;* but the modes of putting nouns and verbs together, express the relations of objects and events, which can be cognised only by the *intellect,* and each different mode corresponds to a different relation. The structure of every sentence is a lesson in logic."

Much more attention should be devoted to explaining the meaning, origin and etymology of words employed. I believe also, that, aside from a few exceptional cases, where it is to be continued through college, the study of Latin should be exclusively along the lines which will bring out its fundamental relation to English.

One special word regarding psychology. It seems to me that the attempt to treat the subject of psychology in one term, is worse than useless. It simply results in getting the children's heads in the clouds, and their feet off terra firma. I believe that a thoughtful teacher will do better untrammeled and naturally, than when trying to follow principles of psychology, imperfectly understood.

Whether one's education is obtained in one way or another should make no difference; whether it be in the graded grammar school, high school and college, or whether it be by the wayside and alone. In fact, without desiring to minimise the real advantages of a good college training, including the atmosphere of learning, culture and refinement, of comradeship and "esprit de corps," more or less inherent, still something can be said for at least an occasional development removed from the curriculum and associations of a university. It has often seemed that one result of regular attendance and continued training from the years of five or six to twenty-two or more is a molding of the intellect which is altogether too uniform, giving a mind seriously lacking in originality and initiative, as well as unfitted, in some cases for practical life.

A noted inventor once explained, in an interesting public address, how he would have failed in certain discoveries and achievements of the greatest practical value, if he had had the ordinary training in chemistry. He would then have been so impressed with the recognised conception of the supposed practical impossibility of his undertaking that he would never have attempted it. As it was, he tried and was successful.

As we all know, it is nothing uncommon to meet persons of unusual intelligence, whose education has been very irregular. This has been true of many of the greatest minds in the world's history. It has frequently happened to me, that upon inquiry as to the source of the education of some one who had impressed me as especially bright and interesting, that I learned to my surprise, how very limited had been the ordinary school attendance. A well known lady of this city, whose command of the English language is certainly second to none, informed me that her instruction in technical grammar was limited to one lesson, given her by her father.

Entrance examinations cannot, of course, be entirely dispensed with. It would be manifestly unjust to allow young men, more or less unprepared to waste several years, only to discover finally, their inability to finish. But while admitting this, it still seems that an improvement might be made upon the present system of Regents' counts.

The question of examinations is a difficult one, but it is certain that, at present, altogether too much stress is laid on memorising, to the very great exclusion of faculties infinitely more important. But even if we limit our consideration to memory, it is certainly true that to understand a matter thoroughly, is the surest and most lasting method of memorising. When a subject is once thoroughly understood, it requires but little effort of memory to make it a permanent acquisition. Simple memory is, as a rule, very evanescent. To be convinced of this, one need only question the average young person, a year or two out of school. But a subject once well comprehended, though temporarily forgotten, is readily recalled to mind by a little thinking.

The practical application, which as medical students, we should make of these facts is to study deliberately, comprehending thoroughly as we proceed, leaving little to pure memory. It would seem, also, that the argument advanced in favor of a course of study which would insure a general acquaintance of the field of knowledge as a whole, with, at the same time, a quite thorough knowledge of a special limited department might equally be applied to the study of medicine itself. What has been said of knowledge as a whole is true even of the limited subject of medicine.

Even medicine in its entirety can no longer be completely mastered by one individual. The best solution of the problem, here also, would seem to be found in an earnest study of the science and art in general, with, at the same time, a fairly profound mastery of some limited part. This plan would apply to general practitioners as well as to specialists. Both would be benefited. Dr. Frank Hamilton in an interesting and witty address once said "many specialists, in addition to their particular scopes, such as the stethoscope, the laryngoscope, the ophthalmoscope, and the like, need still more a wider scope." So, also, it may be said of many a general practitioner that his knowledge should be more definite and exact; that he should be more familiar with the advances in the various specialties. In other words, while his scope or field must necessarily always remain large he should still constantly strive to keep his focus clear and definite.

In conclusion, let us recognise the fact that as physicians, interested both in the mind and the body we are especially called upon to discourage and to actively oppose the present tendency to crowd and to hurry. As citizens of a republic, also, we should particularly recognise the evil effect which this practice has upon public affairs, and upon the community in general.

Herbert Spencer in his "Data of Ethics" speaking of true and false altruism says:

> I do not mean such altruism as taxes rate payers, that children's minds may be filled with dates, and names, and gossip about kings, and narratives of battles, and other useless information, no amount of which will make them capable workers or good citizens; but I mean such altruism as helps to spread a knowledge of the nature of things and to cultivate the power of applying that knowledge.

These words of one of the clearest and most logical minds of this or any age, make a fitting conclusion to an appeal to make our system of instruction the first step in an education that shall be real; an education that shall have less of shell and more of substance, less of show and more of reality; an education that shall have in it the germ of development throughout the life of the individual, that shall make us more thoughtful, more modest, more reverent, more just, more wise.

242 NORWOOD AVE.

